# Characterizing Common Factors Affecting Replication Initiation During H_2_O_2_ Exposure and Genetic Mutation-Induced Oxidative Stress in *Escherichia coli*

**DOI:** 10.3390/ijms26072968

**Published:** 2025-03-25

**Authors:** Jiaxin Qiao, Weiwei Zhu, Dongdong Du, Morigen Morigen

**Affiliations:** 1 Inner Mongolia Key Laboratory for Molecular Regulation of the Cell, School of Life Sciences, Inner Mongolia University, Hohhot 010070, China; jxqiao924@163.com (J.Q.); dudongdong2018@126.com (D.D.); 2 State Key Laboratory of Vaccines for Infectious Diseases, Xiang-An Biomedicine Laboratory, Department of Laboratory Medicine, School of Public Health, Xiamen University, Xiamen 361102, China; 3 State Key Laboratory of Molecular Vaccinology and Molecular Diagnostics, Department of Laboratory Medicine, School of Public Health, Xiamen University, Xiamen 361102, China

**Keywords:** oxidative stress, DNA replication initiation, ATP levels, efflux pumps, metabolism

## Abstract

Oxidative stress is prevalent in organisms, and excessive oxidative damage can trigger cell death. Bacteria have evolved multiple pathways to cope with adverse stress, including the regulation of the cell cycle. Previous studies show that non-lethal exposure to H_2_O_2_ and mutations in antioxidant enzymes suppress replication initiation in *Escherichia coli*. The existence of common regulatory factors governing replication initiation across diverse causes-induced oxidative stress remains unclear. In this study, we utilized flow cytometry to determine the replication pattern of *E. coli*, and found that oxidative stress also participated in the inhibition of replication initiation by a defective iron regulation (*fur*-*bfr*-*dps* deletion). Adding a certain level of ATP promoted replication initiation in various antioxidant enzyme-deficient mutants and the Δ*fur*Δ*bfr*Δ*dps* mutant, suggesting that low ATP levels could be a common factor in the inhibition of replication initiation by different causes-induced oxidative stress. More potential common factors were screened using proteomics, followed by genetic validation with H_2_O_2_ stress. We found that oxidative stress might mediate the inhibition of replication initiation by interfering with the metabolism of glycine, glutamate, ornithine, and aspartate. Blocking CcmA-dependent cytochrome *c* biosynthesis, deleting the efflux pump proteins MdtABCD and TolC, or the arabinose transporter AraFHG eliminated the replication initiation inhibition by H_2_O_2_. In conclusion, this study uncovers a common multifactorial pathway of different causes-induced oxidative stress inhibiting replication initiation. Dormant and persistent bacteria exhibit an arrested or slow cell cycle, and non-lethal oxidative stress promotes their formation. Our findings contribute to exploring strategies to limit dormant and persistent bacterial formation by maintaining faster DNA replication initiation (cell cycle progression).

## 1. Introduction

Bacteria regulate cell cycle processes to survive in adverse environments [1,2]. Reactive oxygen species (ROS) produced by host immune cells can inhibit or kill pathogenic bacteria, but can also lead to the formation of dormant or persistent bacteria. The presence of these two types of bacteria severely reduces the efficacy of clinical anti-infective treatments and results in recurrent infections. Oxidative stress also induces a viable but not culturable (VBNC) state in bacteria. Bacteria in the dormant and VBNC states are typically regarded as being in complete cell cycle arrest. Persistent cells neither grow nor die in response to antibiotic stress, and DNA replication is inhibited [3]. *Escherichia coli* responds to non-lethal oxidative stress by inhibiting replication initiation [4]. This suggests that oxidative stress may promote the development of persistent bacteria by inhibiting replication initiation. Indeed, oxidative stress produced by immune cells prompts *Salmonella* to form antibiotic-persistent cells [5].

VBNC and dormant bacteria populations exhibit pan-stress resistance [6]. Commonly used antibiotics can be significantly less effective against persistent cells and are largely ineffective against VBNC and dormant bacteria [7], suggesting that a slowed cell cycle inhibits antibiotic efficacy. Bacterial resistance is becoming a serious threat to human health [8]. This makes exploring how oxidative stress inhibits replication initiation attractive, which may help develop antibiotic adjuvants that promote DNA replication.

Hoff et al. found that H_2_O_2_ treatment inhibited the initiation of DNA replication in *E. coli* [9]. Manganese ions contribute to the rapid recovery of DNA replication following oxidative stress [10,11]. However, a thorough investigation into the effects of oxidative stress on replication initiation is lacking. In previous work, we determined the inhibitory effect of non-lethal oxidative stress on replication initiation in terms of both H_2_O_2_ exposure and mutations in antioxidant enzymes. Subsequent mechanistic investigations have shown that the base excision repair DNA glycosylase MutY is resistant to the inhibition of replication initiation by H_2_O_2_. Additionally, Lon protease deficiency alleviates inhibition of replication initiation induced by exogenous H_2_O_2_ exposure, but does not affect inhibition resulting from *katE* or *sodA*-*sodB* deletion. Deletions of *clpP* and *hslV* further delay replication initiation in the Δ*katE* mutant, while deletion of *hflK* promotes replication initiation in both the Δ*katE* and Δ*sodA*Δ*sodB* mutants [4], indicating that AAA+ proteases have a flexible role in regulating replication initiation in response to oxidative stress.

Overall, different causes-induced oxidative stress do not similarly inhibit replication initiation. This highlights the importance of characterizing common factors that inhibit replication initiation by different causes-induced oxidative stress. For example, strategies based on common mechanisms can be generally applied to explore molecules based on stimulation of replication initiation for restoration of dormancy, persistence, and VBNC state bacterial susceptibility to antibiotics. In this work, we identified an ensemble consisting of amino acid metabolism, cytochrome *c* synthesis, efflux pumps, the arabinose transporter AraFGH, and ATP levels involved in the regulation of replication initiation by different causes-induced oxidative stress. This provides a new perspective on restoring sensitivity to antibiotics in cells (such as persistent bacteria) whose DNA replication is inhibited by oxidative stress.

## 2. Results

### 2.1. Defective Iron Regulation Mediates Delayed Replication Initiation and Can Be Reversed by the ROS Scavenger NAC

**Figure 1:**
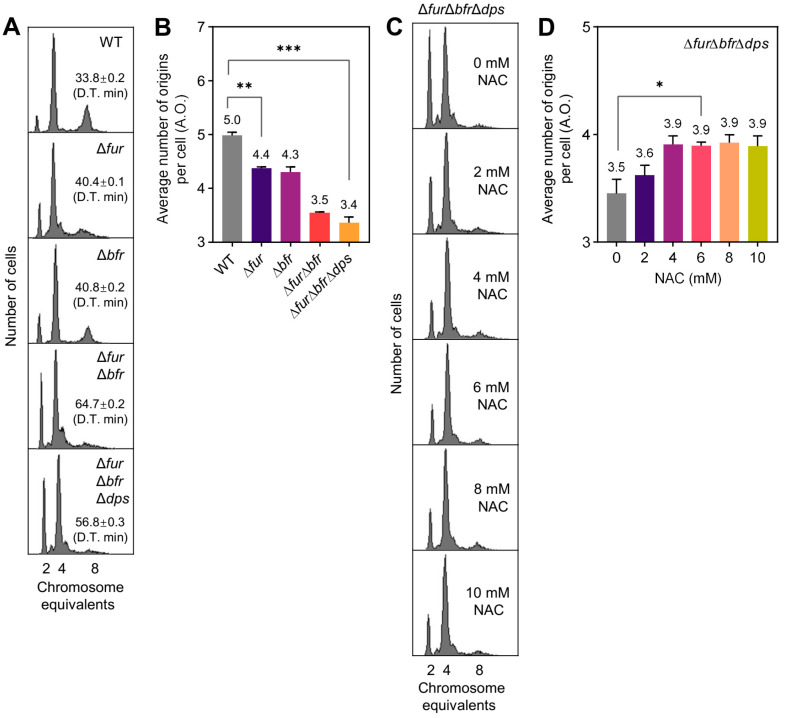
The effect of iron metabolism on replication initiation is associated with oxidative stress. (**A**,**C**) DNA replication patterns of *E. coli*. Exponentially growing *E. coli* cells were cultured to OD_450_ = 0.15–0.2 in ABTG-CAA medium at 37 °C and treated with rifampicin and cephalexin for 3–5 doubling times. *E. coli* cells were fixed with 70% ethanol and then incubated with Hoechst 33,258 fluorescent dye; 10,000 cells were analyzed for DNA replication patterns using flow cytometry. The number of chromosome equivalents contained per cell is indicated on the *X–axis*, and the number of cells is shown on the *Y–axis*. In panel (**A**), doubling times are labeled in boxes and indicated by D.T. In panel (**C**), the indicated concentrations of ATP were added at around OD_450_ = 0.04 and co-incubated with the culture for about two doubling times to grow to OD_450_ = 0.15–0.2. (**B**,**D**) The average number of replication origins contained per bacterial cell in the respective category in panel (**A**,**C**). The average number of replication origins per cell (A.O.) represents the sum of the products of the number of chromosomes and the percentage of related cells. Data are the average of three independent biological replicates (marked above the corresponding column plot and showing one valid digit after the decimal point), and error bars represent standard deviations. Data significance analysis was performed using a *t*-test (two-tailed, sample-paired method). *: 0.01 < *p*-value < 0.05; **: 0.001 < *p*-value < 0.01; ***: *p*-value < 0.001.

Iron regulates replication initiation, while the Fenton reaction, in which divalent iron is involved, produces hydroxyl radicals with a robust oxidizing capacity [11]. Apart from exogenous H_2_O_2_ exposure and defective antioxidant enzymes [4], in this work, we first investigated the effects of iron homeostasis regulators Fur [12] and Bfr [13] on replication initiation to identify more general common factors. The absence of *fur* or *bfr* induced an increase in the number of cells containing two chromosomes and a decrease in the number of cells containing eight chromosomes (Figure 1A). The average number of replication origins per cell (A.O.) in *E. coli* was reduced from 5.0 in BW25113 (wild-type, WT) to 4.4 in ∆*fur* and 4.3 in ∆*bfr* (Figure 1B), indicating a delay in DNA replication initiation. The double deletion of *fur*-*bfr* had a superimposed effect on replication initiation inhibition (Figure 1A,B). Dps is an iron-chelating protein known as a replication initiation repressor [14,15]. To avoid interference from Dps action, we deleted *dps* from the ∆*fur*∆*bfr* mutant. The *fur*-*bfr*-*dps* triple deletion inhibited replication initiation to a comparable extent as the ∆*fur*∆*bfr* mutant (Figure 1A,B). The 6 mM ROS scavenger N-Acetylcysteine (NAC) significantly reversed the delay in replication initiation caused by *fur*-*bfr*-*dps* deletion (Figure 1C,D).This is consistent with data that NAC can reverse the inhibition of replication initiation caused by H_2_O_2_ exposure and antioxidant enzyme defects [4]. Our findings indicate that inhibition of replication initiation due to deletion of iron homeostasis-associated genes is associated with oxidative stress.

### 2.2. ATP Levels Associated with Delayed Replication Initiation Due to Oxidative Stress

**Figure 2:**
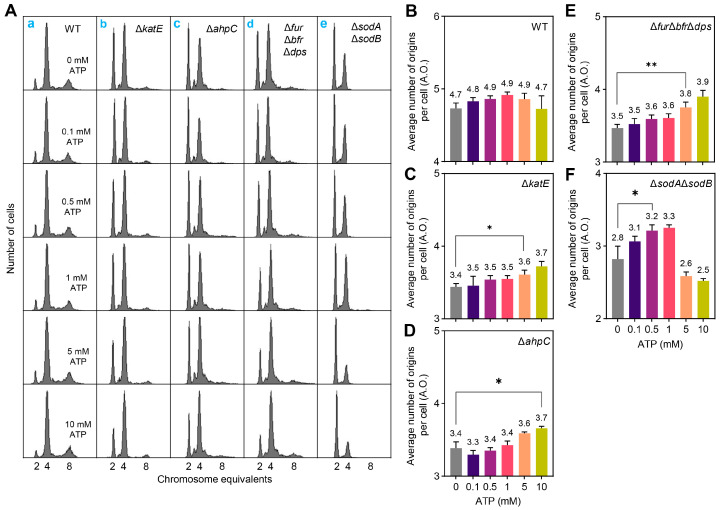
ATP levels associated with the effects of oxidative stress on replication initiation. (**A**) DNA replication patterns of *E. coli*. Exponentially growing *E. coli* cells were cultured to OD_450_ = 0.15–0.2 in ABTG-CAA medium at 37 °C and treated with rifampicin and cephalexin for 3–5 generations (doubling times). Cells were fixed with 70% ethanol and then incubated with Hoechst 33,258 fluorescent dye; 10,000 cells were analyzed for DNA replication patterns by flow cytometry. The number of chromosome equivalents contained per cell is indicated on the *X–axis*, and the number of cells is shown on the *Y–axis*. The indicated concentrations of ATP were added at around OD_450_ = 0.04 and co-incubated with the culture for two doubling times to grow to OD_450_ = 0.15–0.2. (**B**–**F**) The average number of replication origins contained per bacterial cell in the respective category in panel (**A**(**a**–**e**)). The average number of replication origins per cell (A.O.) represents the sum of the products of the number of chromosomes and the percentage of related cells. Data are the average of three independent biological replicates (marked above the corresponding column plot and showing one valid digit after the decimal point), and error bars represent standard deviations. Data significance analysis was performed using a *t*-test (two-tailed, sample-paired method). *: 0.01 < *p*-value < 0.05; **: 0.001 < *p*-value < 0.01.

Oxidative stress decreases intracellular ATP levels [16],and the repair system uses ATP for energy to repair oxidative damage. Since the replication initiation activity of DnaA depends on ATP binding [17], oxidative stress-mediated ATP depletion may reduce the level of the ATP-DnaA complex, which adversely affects replication initiation. To test this idea, we supplemented cultures with exogenous ATP. Adding 0–10 mM ATP did not alter replication initiation in WT (Figure 2A(a),B), but 5 mM or 10 mM ATP promoted replication initiation in the ∆*katE* (catalase HPII), ∆*ahpC* (alkyl hydroperoxide reductase), and ∆*fur*∆*bfr*∆*dps* mutants (Figure 2A(b–d),C–E). Unexpectedly, 0.5–1 mM ATP promoted ∆*sodA*∆*sodB* (superoxide dismutase) mutant replication initiation, but 5–10 mM ATP failed (Figure 2A(e),F), indicating that the effect of high-concentration ATP masked the promotion of replication initiation in the ∆*sodA*∆*sodB* mutant by low-concentration ATP. ATP could be an antioxidant [18]. To avoid unpredictable interference caused by the simultaneous addition of both active molecules, we did not test the effect of exogenous ATP on replication initiation following H_2_O_2_ exposure. Overall, reduced ATP levels serve as a common factor mediating the inhibition of replication initiation caused by different causes-induced oxidative stress.

### 2.3. Proteomic Screening and Genetic Validation of Common Factors That Inhibit Replication Initiation Under Different Causes-Induced Oxidative Stresses

**Figure 3:**
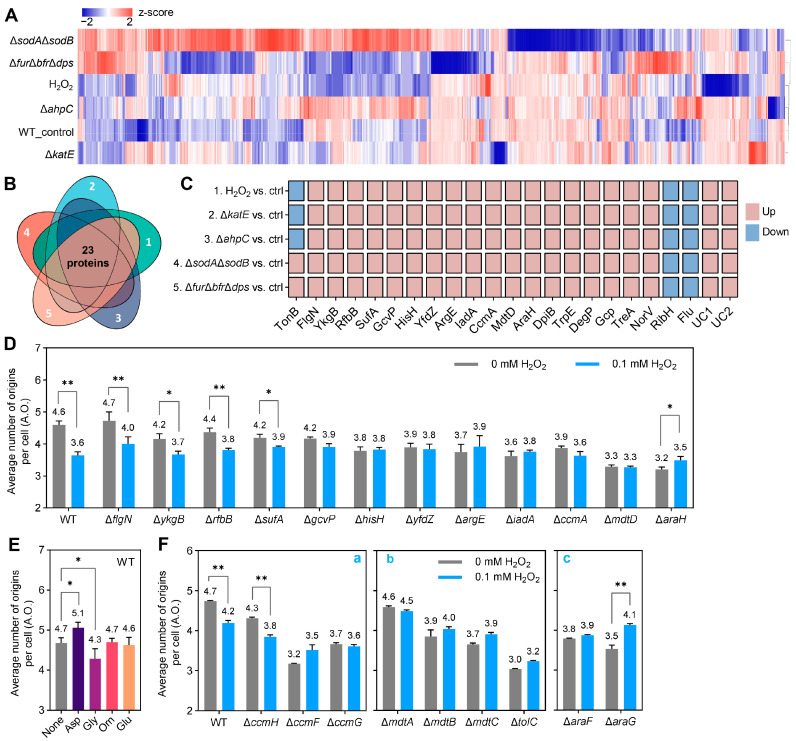
Proteomic screen and genetic validation of common factors affecting replication initiation during oxidative stress. (**A**) Global protein expression profiles. H_2_O_2_, 0.1 mM H_2_O_2_. WT_control, WT without H_2_O_2_ treatment. (**B**) Venn diagram showing the number of proteins that were co-significantly differentially expressed in the various treatments compared to the control. The comparison groups represented by the Arabic numerals correspond to panel (**C**). (**C**) Specific names and expression trends of proteins listed in panel (**B**). UC indicates uncharacterized proteins. ctrl, control (WT without H_2_O_2_ treatment); Up, up-regulated; Down, down-regulated. (**D**,**F**) Effects of the absence of proteomics-screened proteins and their associated proteins on replication initiation under H_2_O_2_ stress. The average number of replication origins per cell (A.O.) represents the sum of the products of the number of chromosomes and the percentage of related cells. The indicated concentrations of H_2_O_2_ were added at around OD_450_ = 0.08 and co-incubated with the culture for one doubling time to grow to OD_450_ = 0.15–0.2. (**E**) Effects of exogenous glycine, glutamate, ornithine, and aspartate on BW25113 (WT) replication initiation. 10 mM glycine (Gly), glutamate (Glu), ornithine (Orn), and aspartate (Asp) were added at around OD_450_ = 0.04 and co-incubated with the culture for two doubling times to grow to OD_450_ = 0.15–0.2. Data are the average of three independent biological replicates (marked above the corresponding column plot and showing one valid digit after the decimal point), and error bars represent standard deviations. Data significance analysis was performed using a *t*-test (two-tailed, sample-paired method). *: 0.01 < *p*-value < 0.05; **: 0.001 < *p*-value < 0.01. Treatments with insignificant differences are not indicated.

Proteins are the executors of biological functions, and we then performed proteomic analyses to unravel additional potential common regulatory factors in addition to ATP. The protein expression profiles of the ∆*katE* and ∆*ahpC* mutants were closer to those of the WT (control), whereas the ∆*sodA*∆*sodB* mutant showed the greatest deviation from WT (Figure 3A). The various treatments resulted in shared differences in the expression of 23 proteins compared with the control (Figure 3B). Further attention was given to proteins whose expression trends were consistent across treatments, thus excluding TonB (Figure 3C). To determine the role of differential expression of these proteins in oxidative stress-mediated replication initiation inhibition, we selected the direct oxidant H_2_O_2_ for subsequent phenotypic validation. Excluding two uncharacterized proteins and unsuccessfully obtained gene deletion strains, we examined the effect of *flgN* and 11 other gene deletions on H_2_O_2_ inhibition of replication initiation (Figure 3D and Figure S1). Replication initiation was still suppressed by 0.1 mM H_2_O_2_ after the deletion of *flgN*, *ykgB*, *rfbB*, and *sufA*, suggesting that these four genes may not be linked to the inhibition of replication initiation by oxidative stress (Figure 3D and Figure S1).

GcvP (depletion of glycine) [19], HisH (generation of glutamate) [20], YfdZ (catalyzes the bidirectional process of glutamate generation and depletion) [21], ArgE (generation of ornithine) [22] and IadA (generation of aspartate) [23] were highly expressed across all treatments (Figure 3C). Deletion of the corresponding genes eliminated the inhibition of replication initiation by H_2_O_2_ (Figure 3D). Adding aspartate promotes WT cells replication initiation (Figure 3E and Figure S2), consistent with previous findings [24]. Additional glycine resulted in delayed replication initiation, whereas glutamate and ornithine had no significant effect on replication initiation in the WT (Figure 3E and Figure S2). These data suggest that under oxidative stress, high expression of IadA for aspartate production can promote replication initiation, as can glycine depletion due to high GcvP expression.

The existence of two branching pathways for cytochrome c biogenesis, dependent on CcmA or CcmH, and the subsequent shared process are carried out by CcmF and CcmG [25]. Defects in *ccmA*, *ccmF*, and *ccmG*, but not *ccmH*, eliminated the inhibition of replication initiation by H_2_O_2_ while inhibiting replication initiation (Figure 3D,F(a) and Figure S3). This suggests that H_2_O_2_ may inhibit replication initiation by disrupting CcmA-dependent cytochrome c biogenesis.

MdtD is a putative multidrug efflux pump protein [26] whose defects inhibited replication initiation and eliminated the inhibition of replication initiation by H_2_O_2_ (Figure 3D). This suggests that H_2_O_2_ may cause functional defects in the efflux pump protein MdtD, leading to the inhibition of replication initiation and inducing compensatory high expression of the MdtD protein. The absence of the efflux pump protein MdtABC, which belongs to the same operon as MdtD, along with the efflux pump common outer membrane channel protein TolC, also eliminated the inhibition of replication initiation by H_2_O_2_ (Figure 3F(b)), suggesting that oxidative stress may inhibit replication initiation by disrupting the efflux pump system.

A deficiency of AraFHG, an arabinose ABC transporter [27], suppressed replication initiation and eliminated the inhibition of replication initiation by H_2_O_2_. Moreover, incubation with H_2_O_2_ following the deletion of *araH* and *araG* promoted replication initiation (Figure 3D,F(c)), suggesting that oxidative stress may lead to a failure of the arabinose transporter and, consequently, inhibit replication initiation.

## 3. Discussion

Regulating cell cycle progression is crucial for maintaining genetic fidelity and ensuring bacterial survival under environmental stress [1,2]. Our recent work focused on how oxidative stress affects the *E. coli* cell cycle [4]. The cell cycle encompasses both replication and division, and the relationship between these events remains an intriguing and contentious topic [28,29,30,31]; division and replication cycles may not even be coupled [32]. Consequently, we aimed to uncover the impact of oxidative stress on the *E. coli* cell cycle, particularly regarding replication initiation. Our previous studies demonstrated that oxidative stress from non-lethal H_2_O_2_ exposure and mutations in antioxidant enzymes inhibits replication initiation, which involves the base excision repair system and the AAA+ protease [4].

The present work firstly found that oxidative stress is also involved in iron regulatory imbalance-mediated inhibition of replication initiation (Figure 1), and mainly explored potential common factors for the inhibition of replication initiation by oxidative stress from different causes (Figure 2 and Figure 3). The response of the efflux pump to oxidative stress is not surprising (Figure 3C, MdtD). As reported, activation of the transcription factor MarA by oxidative stress promotes the expression of the efflux pump ArcA/ArcB [33]. TolC plays a role in bacterial growth under reactive nitrogen species (NO) [34] and oxidative stress [35]. The MacAB efflux pump protects *Salmonella* from oxidative stress [36]. This work emphasized the role of the MdtABCD efflux pump in coordinating oxidative stress and replication initiation (Figure 3D,F(b)).

Bacterial metabolism influences replication initiation [37,38]. Here, we found that the metabolism of glycine, glutamate, ornithine, aspartate, and cytochrome *c* are involved in the replication initiation regulation under oxidative stress (Figure 3D–F). Glycine and glutamate serve as substrates for synthesizing the antioxidant glutathione [39], and glycine also acts as an antioxidant [40]. Glutamate, ornithine, aspartate, and cytochrome *c* have been reported to be associated with oxidative stress [41,42,43], which explains why their related metabolic genes were singled out (Figure 3C). Interestingly, our experimental medium lacked arabinose, but the arabinose transporter protein AraH was prominently tagged (Figure 3C). The expression of the AraFGH operon is positively regulated by the global regulator CRP [44,45], which is important for bacteria resist adverse stress [46,47,48]. This suggests that inhibition of replication initiation by AraFGH may be a general bacterial response to adverse environments, including oxidative stress.

In the above, we described the roles of amino acid metabolism, cytochrome *c* synthesis, efflux pumps, and the arabinose transporter protein AraFHG in inhibiting replication initiation under oxidative stress. Precisely how these factors inhibit replication initiation requires further in-depth study. The key observation is that they all correlate with ATP levels. Metabolism involves energy conversion from ATP; both the efflux pump and the arabinose uptake system AraFGH require ATP for energy [49,50]. Our findings support the idea of ATP as a central molecule connecting oxidative stress to replication initiation: exogenous ATP reversed the delay in replication initiation caused by mutations in antioxidant enzymes and iron metabolism-related genes (Figure 2). While ATP promotes replication initiation in ∆*katE*, ∆*ahpC*, and ∆*fur*∆*bfr*∆*dps* mutants at a concentration of 5 mM, much higher than physiological levels in bacterial cells [51], this could be attributed to the negative charge that ATP carries, which limits the efficiency of exogenous ATP entry into the cell. Additionally, why 0.5–1 mM ATP promoted replication initiation in the ∆*sodA*∆*sodB* mutant but 5–10 mM ATP failed remains unclear. This may be related to the specific oxidative properties of superoxide induced by the *sodA*-*sodB* mutation [52,53,54], which differ from the hydrogen peroxide hydroxyl radicals induced by *katE*, *ahpC*, or *fur*-*bfr*-*dps* mutations.

Overall, we described a multifactorial regulatory framework concerning the inhibition of DNA replication initiation by oxidative stress in *E. coli*. It would be beneficial to investigate the role of these factors in the formation of cell cycle-arrested cells (e.g., oxidative stress-induced dormant bacteria), which may aid in developing antimicrobial strategies aimed at promoting faster cell cycle progression.

## 4. Materials and Methods

### 4.1. Bacterial Strains and Culture

The *Escherichia coli* K-12 strains used in this study are listed in Table S1, and plasmids are listed in Table S2. PCR confirmed the strains from the Keio Collection, with the involved primers listed in Table S3. The Δ*fur* mutant was constructed by homologous recombination via a one-step chromosome gene inactivation method. pCP20 was employed to eliminate resistance genes when necessary [55]. Multi-gene mutant strains were constructed by P1 phage transduction [56]. All strains were incubated overnight in Luria-Bertani medium at 37 °C with shaking at 200 rpm and then diluted at a ratio of 1:5000 in ABTG-Casamino acid (CAA) medium for subsequent experiments. The ABTG-CAA medium consists 6 g/L Na_2_HPO_4_, 2 g/L (NH_4_)_2_SO_4_, 3 g/L KH_2_PO_4_, 3 g/L NaCl, 10 mg/L vitamin B1, 3 μM FeCl_3_, 0.1 mM CaCl_2_, 1 mM MgCl_2_, 0.2% glucose, and 0.5% CAA [57].

### 4.2. Doubling Time Measurement

*E. coli* cells were exponentially grown in ABTG-CAA medium at 37 °C, and the OD_450_ values (0.05–0.5) of the cultures were measured at various time points using an ultraviolet spectrophotometer (UV-1800, Shimadzu, Kyoto, Japan), to calculate the bacterial doubling time [58].

### 4.3. DNA Replication Patterns Determination

Exponentially growing cells in ABTG-CAA medium were treated with 300 μg/mL rifampicin and 10 μg/mL cephalexin monohydrate for 3–5 generations (doubling times) to complete the ongoing replication rounds. Rifampicin and cephalexin monohydrates inhibit replication initiation by blocking transcription and cell division. After washing the cells with 1× TE buffer, they were fixed in 70% ethanol overnight and stored at 4 °C. After replacing TE buffer with Tris-HCl, cells were stained for 30 min in Hoechst 33,258 (Invitrogen, Carlsbad, CA, USA) at a final concentration of 1.5 μg/mL. Then, the replication pattern was analyzed using flow cytometry (Fortesa, BD), with 10,000 cells recorded for each sample. The fluorescence intensity corresponds to different chromosome numbers, and the average number of replication origins per cell (A.O.) was obtained from the formula (A.O. = 2 × proportion of cells containing two chromosomes + 4 × proportion of cells containing four chromosomes + 8 × proportion of cells containing eight chromosomes) [59].

### 4.4. Proteomic Sample Preparation

BW25113, Δ*katE*, Δ*ahpC*, Δ*sodA*Δ*sodB*, and Δ*fur*Δ*bfr*Δ*dps* strains cultured overnight in LB medium were re-diluted in fresh ABTG-CAA medium and cultured to OD_450_ = 0.15 to 0.2. During H_2_O_2_ treatment, 0.1 mM H_2_O_2_ was co-incubated with BW25113 cultures with OD_450_ = 0.08 until OD_450_ = 0.15–0.2. Samples were collected by centrifugation, frozen using liquid nitrogen, and stored at −80 °C before being shipped to Majorbio (Shanghai, China) for total protein extraction, library construction, and proteomic analysis. Three biological replicates of each sample were mixed into one assay sample, and two proteomic assays were performed.

### 4.5. Proteomic Analysis

Total protein extraction. Take out the samples in the frozen state and put them on ice. The samples were suspended in protein lysis buffer (8 M urea, 1% SDS), which included Protease Inhibitor Cocktail (Thermo Fisher Scientific, Waltham, MA, USA) to inhibit protease activity, and the mixture was treated with a high-flux tissue grinding machine 3 times, 40 s each. Then, the mixture was incubated on ice for 30 min, vortex mixed for 5–10 s every 5 min. After centrifugation at 16,000× *g* at 4 °C for 30 min, the concentration of protein from the supernatant collected was determined by the Bicinchoninic acid (BCA) method by BCA Protein Assay Kit (Thermo Fisher Scientific). Protein quantification was performed according to the kit protocol. After protein quantification, SDS-PAGE electrophoresis was performed.

Protein digestion. 100 μg protein was re-suspended with Triethylammonium bicarbonate buffer (TEAB) and had a final concentration of 100 mM. The mixture was reduced with Tris (2-carboxyethyl) phosphine (TCEP) with a final concentration of 10 mM at 37 °C for 60 min and alkylated with iodoacetamide (IAM), with a final concentration of 40 mM at room temperature for 40 min in darkness. After centrifugation at 10,000× *g* at 4 °C for 20 min, the pellet was collected, which was re-suspended with 100 μL Triethylammonium bicarbonate buffer (TEAB), which had a final concentration of 100 mM. Trypsin was added at a 1:50 trypsin-to-protein mass ratio and incubated at 37 °C overnight.

Peptide desalting and quantification. After trypsin digestion, the peptides were drained by a vacuum pump. Then, the enzymatically drained peptides were re-solubilized with 0.1% trifluoroacetic acid (TFA), and the peptides were desalted with HLB and drained by a vacuum concentrator. Finally, the peptides were quantified using the Thermo Fisher Scientific Peptide Quantification Kit (item #23275).

DIA mass detection. Equal amounts of peptides were drained in a vacuum centrifuge concentrator. Then, the enzymatically drained peptides were resolubilized with 0.1% trifluoroacetic acid (TFA), and the peptides were desalted with HLB and drained by a vacuum concentrator. Finally, the peptides were quantified using the Thermo Fisher Scientific Peptide Quantification Kit (item #23275). Based on peptide quantification results, the peptides were redissolved in spectrometry loading buffer (2% ACN with 0.1% formic acid) which included appropriate iRT peptide which was used to calibrate retention time and analyzed by an EASY-nLC system (Thermo, USA) coupled with a timsTOF Pro2 mass spectrometer (Bruker, Ettlingen, Germany) at Majorbio Bio-Pharm Technology Co., Ltd. (Shanghai, China). Briefly, the C18-reversed phase column (75 μm × 25 cm, Ionopticks, Little Rock, AR, USA) was equilibrated with solvent A (2% ACN with 0.1% formic acid) and solvent B (80% ACN with 0.1% formic acid). The peptides were eluted using the following gradient: 0–45 min, 3–28% B; 45–50 min, 28–44% B; 50–55 min, 44–90% B; 55–60 min, maintain 90% B. The tryptic peptides were separated at a flow rate of 250 nL/min.

Data-independent acquisition (DIA) data were acquired using a timsTOF Pro2 mass spectrometer operated in DIA-PASEF mode. MS data were collected over an m/z range of 100 to 1700 and an ion mobility range of 0.76 to 1.29 Vs·cm^−2^. Both accumulation time and ramp time were set to 100 ms. During MS/MS data collection, each time cycle contained one MS and ten PASEF MS/MS scans. Exclusion was active after 0.4 min. A total of 14 DIA-PASEF windows were used (25 Th isolation windows).

Protein identification. SpectronautTM (v14.0.200409.43655, Biognosys) software was used to search the DIA-PASEF raw data. Retention times were corrected by iRT, and 6 peptides per protein and 3 daughter ions per peptide were selected for quantitative analysis. The parameters are as follows: Protein FDR ≤ 0.01, Peptide FDR ≤ 0.01, Peptide Confidence ≥ 99%, XIC width ≤ 75ppm. The shared and modified peptides were excluded, and the peak areas were calculated and summed to give the quantitative results. Only the proteins with at least one unique peptide were used for protein identification.

Statistical analyses. Bioinformatic proteomic data analysis was performed with the Majorbio Cloud platform (https://cloud.majorbio.com, accessed on 10 October 2022). *p*-values and Fold change (FC) for the proteins between the two groups were calculated using the R package “*t*-test.” The thresholds of fold change (>1.2 or <0.83) and *p*-value < 0.05 were used to identify differentially expressed proteins (DEPs). All identified proteins were functionally annotated using GO (http://geneontology.org, accessed on 18 September 2021 and the KEGG pathway (http://www.genome.jp/kegg, accessed on 1 September 2021).

## Data Availability

Data supporting the findings of this study are shown in the main text and Supplementary Materials. The proteomics data have been deposited at the integrated proteome resources (https://www.iprox.org, accessed on 1 October 2024) with the ID IPX0009699000 (ProteomeXchange ID: PXD055835).

## Supplementary Materials

The following supporting information can be downloaded at: https://www.mdpi.com/article/10.3390/ijms26072968/s1.

## Abbreviations

The following abbreviations are used in this manuscript:

ROS	reactive oxygen species
VBNC	viable but not culturable
NAC	N-Acetylcysteine
CAA	Casamino acid
A.O.	average number of replication origins per cell
WT	wild-type
