# Supplementary material for: Characterizing Common Factors Affecting Replication Initiation During H2O2 Exposure and Genetic Mutation-Induced Oxidative Stress in Escherichia coli
Source: Int J Mol Sci. 2025 Mar 25;26(7):2968. doi: 10.3390/ijms26072968 (PMC11989076; doi:10.3390/ijms26072968)
Supplement: Supplementary file 1 [file ijms-26-02968-s001.zip › ijms-3508372-supplementary.pdf]

Supplementary Information

Characterizing Common Factors Affecting Replication Initiation During H_2O_2 Exposure and Genetic Mutation-Induced Oxidative Stress in *Escherichia coli*

Jiaxin Qiao [1,†], Weiwei Zhu [2,3,†,*], Dongdong Du[1], Morigen[1,*]

[1] Inner Mongolia Key Laboratory for Molecular Regulation of the Cell, School of Life Sciences, Inner Mongolia University, Hohhot 010070, China.

[2] State Key Laboratory of Vaccines for Infectious Diseases, Xiang-An Biomedicine Laboratory, Department of Laboratory Medicine, School of Public Health, Xiamen University, Xiamen 361102, China.

[3] State Key Laboratory of Molecular Vaccinology and Molecular Diagnostics, Department of Laboratory Medicine, School of Public Health, Xiamen University, Xiamen 361102, China.

This supplementary file includes the following:

1. Figures S1-S3;
2. Tables S1-S3;
3. Supplementary References.

Supplementary Figures

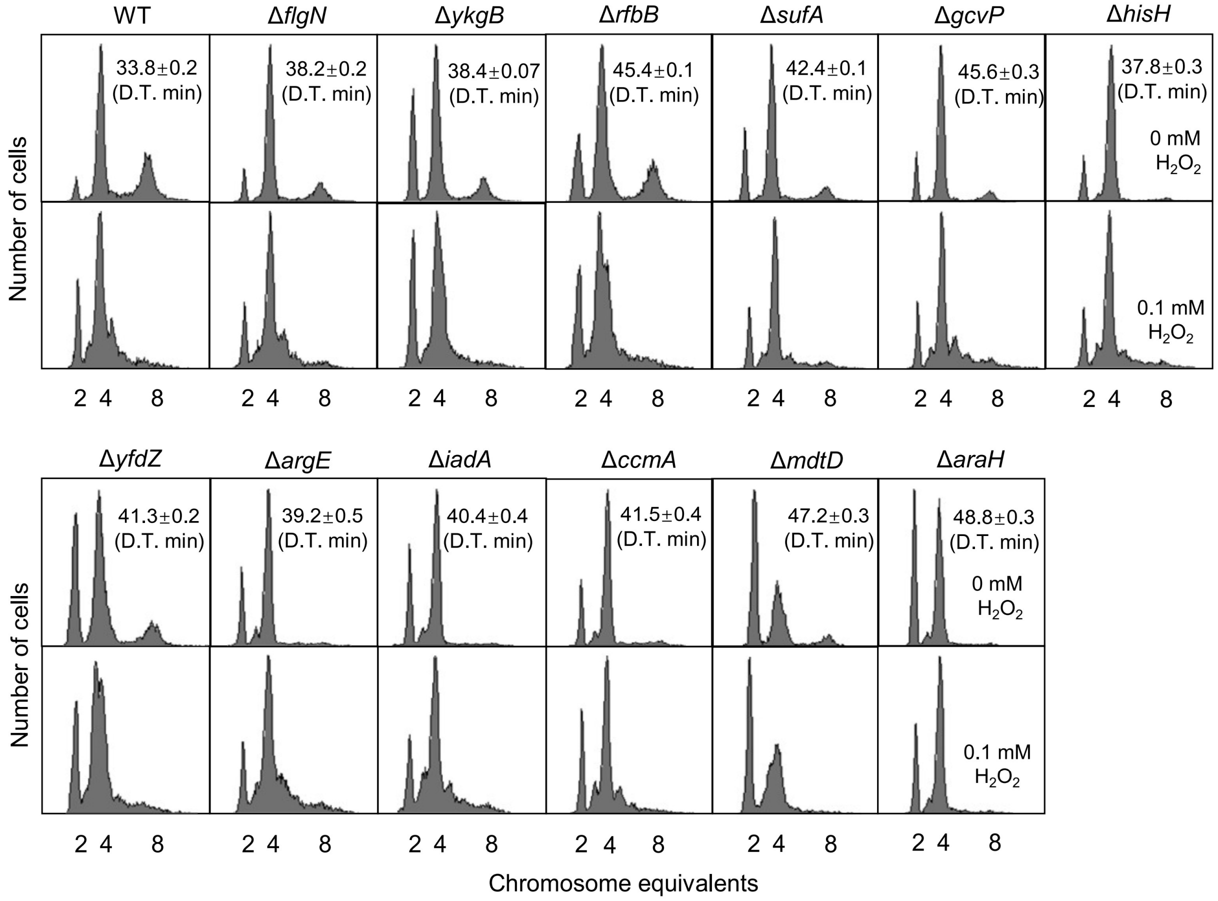

Figure S1. Effects of the absence of proteomics-screened proteins on replication pattern under H_2O_2 stress. Exponentially growing *E. coli* cells were cultured to OD_{450}=0.15-0.2 in ABTG-CAA medium at 37°C and treated with rifampicin and cephalexin for 3-5 doubling times. *E. coli* cells were fixed with 70% ethanol and then incubated with Hoechst 33258 fluorescent dye; 10,000 cells were analyzed for DNA replication patterns by flow cytometry. The number of chromosome equivalents contained per cell is indicated on the X-axis, and the number of cells is shown on the Y-axis. The indicated concentrations of H_2O_2 were added at around OD_{450}=0.08 and co-incubated with the culture for one doubling time to grow to OD_{450}=0.15-0.2. Doubling times are labeled in boxes and indicated by D.T.

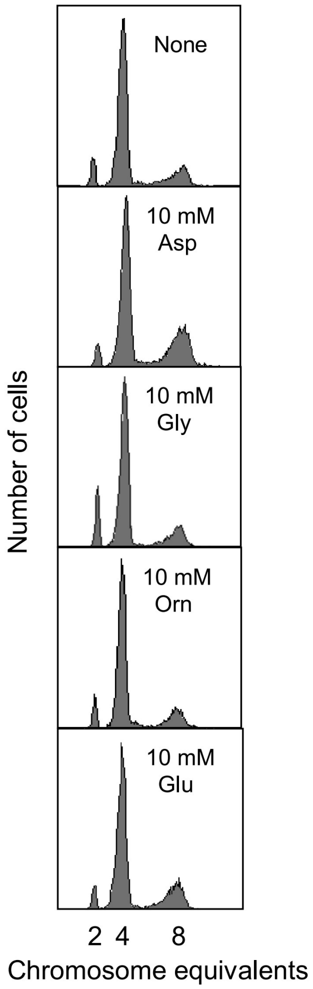

Figure S2. Effects of exogenous glycine, glutamate, ornithine, and aspartate on BW25113 replication pattern. Exponentially growing *E. coli* cells were cultured to OD_{450}=0.15-0.2 in ABTG-CAA medium at 37°C and treated with rifampicin and cephalexin for 3-5 doubling times. 10 mM glycine (Gly), glutamate (Glu), ornithine (Orn), and aspartate (Asp) were added at around OD_{450}=0.04 and co-incubated with the culture for two doubling times to grow to OD_{450}=0.15-0.2. *E. coli* cells were fixed with 70% ethanol and then incubated with Hoechst 33258 fluorescent dye; 10,000 cells were analyzed for DNA replication patterns by flow cytometry. The number of chromosome equivalents contained per cell is indicated on the X-axis, and the number of cells is shown on the Y-axis.

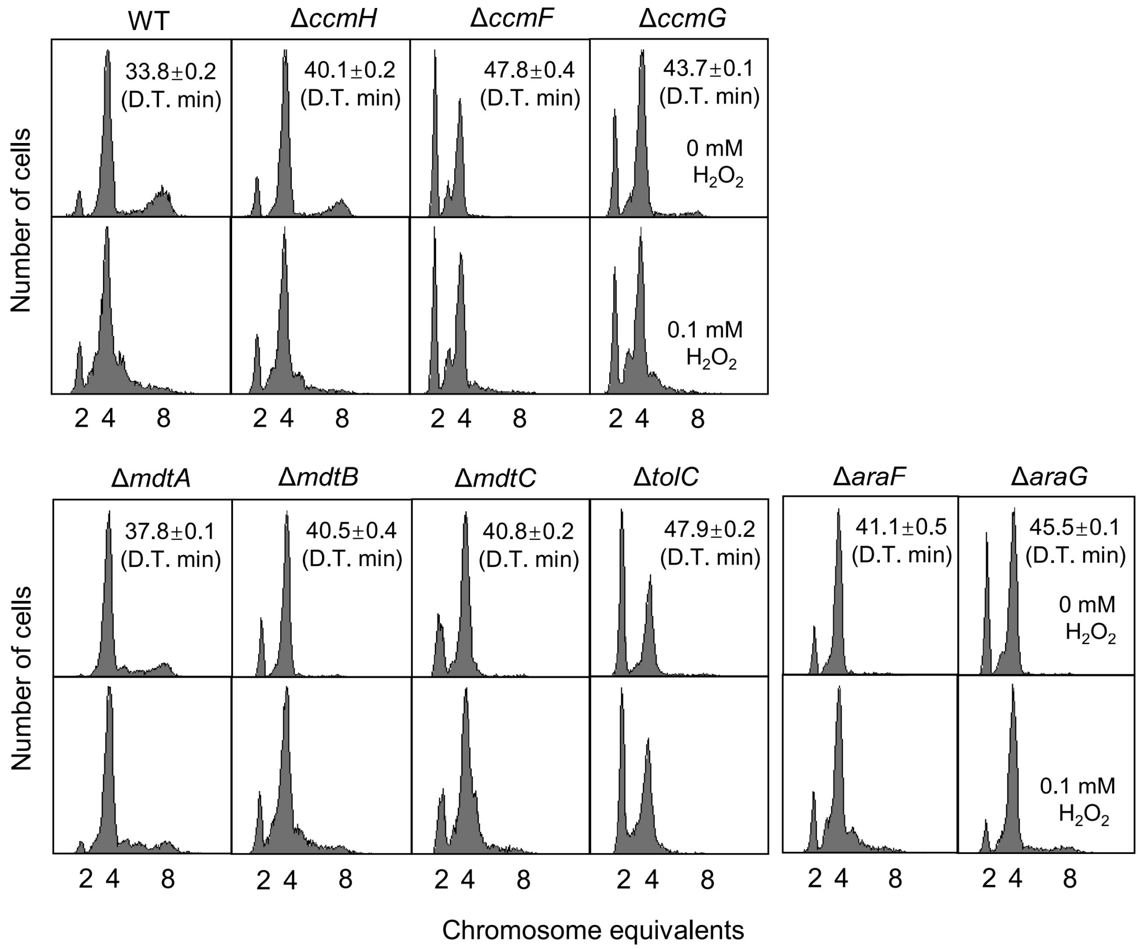

Figure S3. Effects of the absence of proteomics screened protein-associated proteins on replication pattern under H_2O_2 stress. Exponentially growing *E. coli* cells were cultured to OD_{450}=0.15-0.2 in ABTG-CAA medium at 37°C and treated with rifampicin and cephalexin for 3-5 doubling times. *E. coli* cells were fixed with 70% ethanol and then incubated with Hoechst 33258 fluorescent dye; 10,000 cells were analyzed for DNA replication patterns by flow cytometry. The number of chromosome equivalents contained per cell is indicated on the X-axis, and the number of cells is shown on the Y-axis. The indicated concentrations of H_2O_2 were added at around OD_{450}=0.08 and co-incubated with the culture for one doubling time to grow to OD_{450}=0.15-0.2. Doubling times are labeled in boxes and indicated by D.T.

Supplementary Tables

Table S1. Bacterial strains used in the study.

Strains	Genotype	Source or reference
BW25113	*E. coli* wild-type *rrnB*3 Δ*lacZ*4787 *hsdR*514 Δ(*araBAD*)567 Δ(*rhaBAD*)568 *rph-1*	[1]
Δ*fur*	BW25113 Δ*fur*::*cam*^{R}	This work
Δ*bfr*	BW25113 Δ*bfr*::*kan*^{R} Keio Collection	[1]
Δ*dps*	BW25113 Δ*dps*::*kan*^{R} Keio Collection	[1]
Δ*fur*Δ*bfr*	knock out *fur* gene on Δ*bfr* by P1 transduction	This work
Δ*fur*Δ*bfr*Δ*dps*	knock out *dps* gene on Δ*fur*Δ*bfr* by P1 transduction	This work
Δ*sodA*	BW25113 Δ*sodA*::*kan*^{R} Keio Collection	[1]
Δ*sodB*	BW25113 Δ*sodB*::*kan*^{R} Keio Collection	[1]
Δ*sodA*Δ*sodB*	knock out *sodB* gene on Δ*sodA* by P1 transduction	This work
Δ*katE*	BW25113 Δ*katE*::*kan*^{R} Keio Collection	[1]
Δ*ahpC*	BW25113 Δ*ahpC*::*kan*^{R} Keio Collection	[1]
Δ*flgN*	BW25113 Δ*flgN*::*kan*^{R} Keio Collection	[1]
Δ*ykgB*	BW25113 Δ*ykgB*::*kan*^{R} Keio Collection	[1]
Δ*rfbB*	BW25113 Δ*rfbB*::*kan*^{R} Keio Collection	[1]
Δ*sufA*	BW25113 Δ*sufA*::*kan*^{R} Keio Collection	[1]
Δ*gcvP*	BW25113 Δ*gcvP*::*kan*^{R} Keio Collection	[1]
Δ*hisH*	BW25113 Δ*hisH*::*kan*^{R} Keio Collection	[1]
Δ*yfdZ*	BW25113 Δ*yfdZ*::*kan*^{R} Keio Collection	[1]
Δ*argE*	BW25113 Δ*argE*::*kan*^{R} Keio Collection	[1]
Δ*iadA*	BW25113 Δ*iadA*::*kan*^{R} Keio Collection	[1]
Δ*ccmA*	BW25113 Δ*ccmA*::*kan*^{R} Keio Collection	[1]
Δ*ccmH*	BW25113 Δ*ccmH*::*kan*^{R} Keio Collection	[1]
Δ*ccmF*	BW25113 Δ*ccmF*::*kan*^{R} Keio Collection	[1]
Δ*ccmG*	BW25113 Δ*ccmG*::*kan*^{R} Keio Collection	[1]
Δ*mdtD*	BW25113 Δ*mdtD*::*kan*^{R} Keio Collection	[1]
Δ*mdtA*	BW25113 Δ*mdtA*::*kan*^{R} Keio Collection	[1]
Δ*mdtB*	BW25113 Δ*mdtB*::*kan*^{R} Keio Collection	[1]
Δ*mdtC*	BW25113 Δ*mdtC*::*kan*^{R} Keio Collection	[1]
Δ*tolC*	BW25113 Δ*tolC*::*kan*^{R} Keio Collection	[1]
Δ*araH*	BW25113 Δ*araH*::*kan*^{R} Keio Collection	[1]
Δ*araF*	BW25113 Δ*araF*::*kan*^{R} Keio Collection	[1]
Δ*araG*	BW25113 Δ*araG*::*kan*^{R} Keio Collection	[1]

Table S2. Plasmids used in the study.

Plasmids	Genotype	Source or reference
pKD3	*rep*$_{R6K}$ *bla* FRT *cat* FRT	[2]
pKD46	*rep*$_{pSC101}$^{ts}*bla* P_{araBAD} γβ exo	[2]
pCP20	*rep*$_{pSC101}$^{ts}*bla cat cI*857P_R	[2]

Table S3. Primers used in the study.

Primer Name	Sequence （5′--3′）	Usage
check-*sodA*-F	CGCCGTTGTCGATTTACTGG	Upstream primer for Δ*sodA* identification
check-*sodA*-R	ATACGCCTCATTGCAGCAGG	Downstream primer for Δ*sodA* identification
check-*sodB*-F	GATCATGTCGGCGTGTATGT	Upstream primer for Δ*sodB* identification
check-*sodB*-R	ATACAGCTTTGTGCCGGTGA	Downstream primer for Δ*sodB* identification
check-*fur*-F	CTGATGTGATGCGGCGTAGA	Upstream primer for Δ*fur* identification
check-*fur*-R	GTTTGCCGACCAGGAAAGTG	Downstream primer for Δ*fur* identification
check-*bfr*-F	TCGGCAATCAGTGCGGTAAA	Upstream primer for Δ*bfr* identification
check-*bfr*-R	TGGGGCGTCTGGTTAGTTTG	Downstream primer for Δ*bfr* identification
check-*dps*-F	TCCGAAAATTCCTGGCGAGC	Upstream primer for Δ*dps* identification
check-*dps*-R	TGGGCTGCAAAACAAAACGG	Downstream primer for Δ*dps* identification
check-*katE*-F	AAACAGCGGCCCTTTCAGTA	Upstream primer for Δ*katE* identification
check-*katE*-R	AACCTTGCATCCGGCGATTA	Downstream primer for Δ*katE* identification
check-*ahpC*-F	GGCGGTGCAAAGTTCACAAA	Upstream primer for Δ*ahpC* identification
check-*ahpC*-R	AACATCATCATGCAAGCGGC	Downstream primer for Δ*ahpC* identification
check-*flgN*-F	TCTGAAGCCTGTAAGCACCG	Upstream primer for Δ*flgN* identification
Check-*flgN*-R	TTTCTGTTGCGTCTGGTGGT	Downstream primer for Δ*flgN* identification
check-*ykgB*-F	GCATCACCGACAGTTCTGGA	Upstream primer for Δ*ykgB* identification
check-*ykgB*-R	GCCTGACGCCTGAAAAAGTG	Downstream primer for Δ*ykgB* identification
check-*rfbB*-F	TGGGTAACGCTCGTCACATC	Upstream primer for Δ*rfbB* identification
check-*rfbB*-R	CCAACCTACCTGCCCTGTTT	Downstream primer for Δ*rfbB* identification
check-*sufA*-F	TAGCGTGCCTGTTAACCCAC	Upstream primer for Δ*sufA* identification
check-*sufA*-R	ATGCGCGATAGGCGTTTAGA	Downstream primer for Δ*sufA* identification
check-*gcvP*-F	ACGTTTCAGGAACCATCGCT	Upstream primer for Δ*gcvP* identification
check-*gcvP*-R	GACTGCCACCAGTCACAAGT	Downstream primer for Δ*gcvP* identification
check-*hisH*-F	GCTGGATATCTCTGGTCGCC	Upstream primer for Δ*hisH* identification
check-*hisH*-R	TAACGCCGCCACATCTTCTT	Downstream primer for Δ*hisH* identification
check-*yfdZ*-F	CCGGGCGCTAACGTTAATCT	Upstream primer for Δ*yfdZ* identification
check-*yfdZ*-R	GAATTCTGGGGTGATGCCCA	Downstream primer for Δ*yfdZ* identification
check-*argE*-F	TGCGCTGAAACAGTCAAAGC	Upstream primer for Δ*argE* identification
check-*argE*-R	TCGCCCCAAGTAACACCAAA	Downstream primer for Δ*argE* identification
check-*iadA*-F	GTGGGGATTACCCGGTGAAG	Upstream primer for Δ*iadA* identification
check-*iadA*-R	AACACCAATCGCCTGTTCCA	Downstream primer for Δ*iadA* identification

check-*ccmA*-F	TATGCGTGAAGTCGAGCCAG	Upstream primer for Δ*ccmA* identification
check-*ccmA*-R	CGCAGCAACCCAGATAATGC	Downstream primer for Δ*ccmA* identification
check-*ccmH*-F	TACGCGCTAAGCCTGTTTGA	Upstream primer for Δ*ccmH* identification
check-*ccmH*-R	CCGGCATAAACTAAGCGCAC	Downstream primer for Δ*ccmH* identification
check-*ccmF*-F	GCTGAAGGCTCAGTGGATGT	Upstream primer for Δ*ccmF* identification
check-*ccmF*-R	CCTGCGCAGAAAGCTGATTC	Downstream primer for Δ*ccmF* identification
check-*ccmG*-F	TATCGTAAGCGCGTGAGTCC	Upstream primer for Δ*ccmG* identification
check-*ccmG*-R	GCAATGCTGTTGTTCTGGCA	Downstream primer for Δ*ccmG* identification
check-*mdtD*-F	GCCAGCTCCTTACGCTGTAT	Upstream primer for Δ*mdtD* identification
check-*mdtD*-R	AATGCAGGTGGCGAAAATCG	Downstream primer for Δ*mdtD* identification
check-*mdtA*-F	TTCGCCATGCAAGCAGTCTA	Upstream primer for Δ*mdtA* identification
check-*mdtA*-R	CGGAACTTTGCGACGACATC	Downstream primer for Δ*mdtA* identification
check-*mdtB*-F	TGCAAATGGGCAATGAAGGC	Upstream primer for Δ*mdtB* identification
check-*mdtB*-R	CGCTGACGATAATCACCGGA	Downstream primer for Δ*mdtB* identification
check-*mdtC*-F	TATTTGCTGTTCGACCGCCT	Upstream primer for Δ*mdtC* identification
check-*mdtC*-R	AGCACGATGGCGGTAAAGAA	Downstream primer for Δ*mdtC* identification
check-*tolC*-F	ACGTAACGCCAACCTTTTGC	Upstream primer for Δ*tolC* identification
check-*tolC*-R	TTTCACAGCCAGCCAGCATA	Downstream primer for Δ*tolC* identification
check-*araH*-F	AGCCTTGCGATGCCTAAAGT	Upstream primer for Δ*araH* identification
check-*araH*-R	GTTTGATTTCCGCCAGCGTT	Downstream primer for Δ*araH* identification
check-*araF*-F	TGCCCTACACAAAACGACACT	Upstream primer for Δ*araF* identification
check-*araF*-R	ATCACTGGTCGTTCACACCC	Downstream primer for Δ*araF* identification
check-*araG*-F	AGCAAAAGACGTTGAACCGC	Upstream primer for Δ*araG* identification
check-*araG*-R	CACCATCCCCGACATGGAAA	Downstream primer for Δ*araG* identification
del-*fur*-F	GTCACTTCTTCTAATGAAGTGAACC GCTTAGTAACAGGACAGATTCCGCG TGTAGGCTG GAGCTGCTTC	To delete the *fur* gene from chromosome
del-*fur*-R	CTTGCATAAAAAAGCCAACCCGCAG GTTGGCTTTTCTCGTTCAGGCTGGCC ATATGAAT ATCCTCCTTA	To delete the *fur* gene from chromosome

Supplementary References

1. Baba, T.; Ara, T.; Hasegawa, M.; Takai, Y.; Okumura, Y.; Baba, M.; Datsenko, K.A.; Tomita, M.; Wanner, B.L.; Mori, H. Construction of *Escherichia coli* K-12 in-frame, single-gene knockout mutants: the Keio collection. *Mol Syst Biol* **2006**, *2*, 2006.0008, doi:10.1038/msb4100050.
2. Datsenko, K.A.; Wanner, B.L. One-step inactivation of chromosomal genes in *Escherichia coli* K-12 using PCR products. *Proc Natl Acad Sci U S A* **2000**, *97*, 6640-6645, doi:10.1073/pnas.120163297.
